# Supplementary material for: Optogenetic activation of dorsal raphe serotonin neurons induces brain-wide activation
Source: Nat Commun. 2024 May 16;15:4152. doi: 10.1038/s41467-024-48489-6 (PMC11099070; doi:10.1038/s41467-024-48489-6)
Supplement: Supplementary file 3 — Reporting Summary [file 41467_2024_48489_MOESM3_ESM.pdf]

# nature portfolio

Corresponding author(s): Hiro Taiyo Hamada; Kenji Doya

Last updated by author(s): Apr 10, 2024

# Reporting Summary

Nature Portfolio wishes to improve the reproducibility of the work that we publish. This form provides structure for consistency and transparency in reporting. For further information on Nature Portfolio policies, see our Editorial Policies and the Editorial Policy Checklist.

## Statistics

For all statistical analyses, confirm that the following items are present in the figure legend, table legend, main text, or Methods section.

| n/a | Confirmed | |
|---|---|---|
| ☐ | ☒ | The exact sample size (*n*) for each experimental group/condition, given as a discrete number and unit of measurement |
| ☐ | ☒ | A statement on whether measurements were taken from distinct samples or whether the same sample was measured repeatedly |
| ☐ | ☒ | The statistical test(s) used AND whether they are one- or two-sided<br>*Only common tests should be described solely by name; describe more complex techniques in the Methods section.* |
| ☐ | ☒ | A description of all covariates tested |
| ☐ | ☒ | A description of any assumptions or corrections, such as tests of normality and adjustment for multiple comparisons |
| ☐ | ☒ | A full description of the statistical parameters including central tendency (e.g. means) or other basic estimates (e.g. regression coefficient) AND variation (e.g. standard deviation) or associated estimates of uncertainty (e.g. confidence intervals) |
| ☐ | ☒ | For null hypothesis testing, the test statistic (e.g. *F*, *t*, *r*) with confidence intervals, effect sizes, degrees of freedom and *P* value noted<br>*Give P values as exact values whenever suitable.* |
| ☒ | ☐ | For Bayesian analysis, information on the choice of priors and Markov chain Monte Carlo settings |
| ☒ | ☐ | For hierarchical and complex designs, identification of the appropriate level for tests and full reporting of outcomes |
| ☐ | ☒ | Estimates of effect sizes (e.g. Cohen's *d*, Pearson's *r*), indicating how they were calculated |

*Our web collection on statistics for biologists contains articles on many of the points above.*

## Software and code

Policy information about availability of computer code

| | |
|---|---|
| Data collection | Paravision 6 |
| Data analysis | Statistical Parametric Mapping 12(SPM12, https://www.fil.ion.ucl.ac.uk/spm)<br>MarsBaR region of interest toolbox for SPM (http://marsbar.sourceforge.net/)<br>MRIcroGL (https://www.nitrc.org/projects/mricrogl/)<br>Probabilistic Threshold-free Cluster Enhancement (pTFCE, https://spisakt.github.io/pTFCE/)<br>Allen Brain Atlas (https://portal.brain-map.org/)<br>Templates for In Vivo Mouse Brain (https:// www.nitrc.org/ projects/tpm_mouse)<br>MATLAB2020b (https://mathworks.com/)<br>A CBV opfMRI dataset (https://doi.org/10.34973/raa0-5z29) |

For manuscripts utilizing custom algorithms or software that are central to the research but not yet described in published literature, software must be made available to editors and reviewers. We strongly encourage code deposition in a community repository (e.g. GitHub). See the Nature Portfolio guidelines for submitting code & software for further information.

## Data

Policy information about availability of data

All manuscripts must include a data availability statement. This statement should provide the following information, where applicable:

- Accession codes, unique identifiers, or web links for publicly available datasets
- A description of any restrictions on data availability
- For clinical datasets or third party data, please ensure that the statement adheres to our policy

Study data are available in BIDS format at the Openneuro.org Data Repository (Project_ID: optofMRI_DRN).

## Research involving human participants, their data, or biological material

Policy information about studies with human participants or human data. See also policy information about sex, gender (identity/presentation), and sexual orientation and race, ethnicity and racism.

| | |
|---|---|
| Reporting on sex and gender | n/a |
| Reporting on race, ethnicity, or other socially relevant groupings | n/a |
| Population characteristics | n/a |
| Recruitment | n/a |
| Ethics oversight | n/a |

Note that full information on the approval of the study protocol must also be provided in the manuscript.

# Field-specific reporting

Please select the one below that is the best fit for your research. If you are not sure, read the appropriate sections before making your selection.

☒ Life sciences ☐ Behavioural & social sciences ☐ Ecological, evolutionary & environmental sciences

For a reference copy of the document with all sections, see nature.com/documents/nr-reporting-summary-flat.pdf

# Life sciences study design

All studies must disclose on these points even when the disclosure is negative.

| | |
|---|---|
| Sample size | The sample size is determined with R based on previous work [GrandJean et al., 2019].<br><br>power.t.test(delta=0.1, sd=0.05, power=0.8, sig.level=0.05, alternative=two.sided)<br>n = 5.09<br><br>Reference:<br>Grandjean, J., Corcoba, A., Kahn, M.C. et al. A brain-wide functional map of the serotonergic responses to acute stress and fluoxetine. Nat Commun 10, 350 (2019). https://doi.org/10.1038/s41467-018-08256-w |
| Data exclusions | Two mice were excluded from third and fourth sessions the study since a head fixation bar was removed. |
| Replication | In order to check reproducibility of the experiments, functional maps and correlation between responses of target regions of interests (ROIs) by photostimulation in transgenic mice under awake state were compared. A brain response by photostimulation under awake state (session1) was reproduced in session 2 and session 4. Brain responses of target 28 ROIs in session 1 were reproduced these in session 2. These findings indicate that blue photostimulation of DRN serotonin neurons consistently induced similar patterns of brain responses. |
| Randomization | This study is to check brain response by optogenetic stimulations with transgenic mice and wild-type mice. Randamization is not applicable since the mice were assigned to their groups based on their genotype (transgenic or wild-type), and all mice underwent the same stimulation protocols including yellow illumination as a negative control to ensure a controlled experiment. |
| Blinding | In order to ensure an adequate number of samples in each experimental group, the investigator, who is in charge of data collection, was not blinded to the group allocation. However, to minimize potential bias, all investigators were blinded during the data analysis. |

# Reporting for specific materials, systems and methods

We require information from authors about some types of materials, experimental systems and methods used in many studies. Here, indicate whether each material, system or method listed is relevant to your study. If you are not sure if a list item applies to your research, read the appropriate section before selecting a response.

## Materials & experimental systems

| n/a | Involved in the study |
|---|---|
| ☒ | ☐ Antibodies |
| ☒ | ☐ Eukaryotic cell lines |
| ☒ | ☐ Palaeontology and archaeology |
| ☐ | ☒ Animals and other organisms |
| ☒ | ☐ Clinical data |
| ☒ | ☐ Dual use research of concern |
| ☒ | ☐ Plants |

## Methods

| n/a | Involved in the study |
|---|---|
| ☒ | ☐ ChIP-seq |
| ☒ | ☐ Flow cytometry |
| ☐ | ☒ MRI-based neuroimaging |

# Animals and other research organisms

Policy information about studies involving animals; ARRIVE guidelines recommended for reporting animal research, and Sex and Gender in Research

| | |
|---|---|
| Laboratory animals | Eight transgenic and six wild-type (WT) C57BL/6J male mice (age > 12 w.o.) were used in this study. The transgenic mice, serotonin neuron-specific channelrhodopsin 2 variant (C128S), were generated by crossing Tph2-tTA mice with tetO-ChR(C128S)-EYFP knock-in mice under control of the tryptophan hydroxylase 2 (Tph2) promoter. All mice are housed at 24 ◦C on an inverted dark:light cycle (lights on 10:00-22:00, GMT+9). |
| Wild animals | No wild animals were used in this study. |
| Reporting on sex | male |
| Field-collected samples | No field collected samples were used in the study. |
| Ethics oversight | All experiments under the ethical committee by Okinawa Institute of Science and Technology. |

Note that full information on the approval of the study protocol must also be provided in the manuscript.

# Plants

| | |
|---|---|
| Seed stocks | a/n |
| Novel plant genotypes | a/n |
| Authentication | a/n |

# Magnetic resonance imaging

## Experimental design

| | |
|---|---|
| Design type | block design |
| Design specifications | 5 blocks per run spaced 120, 2 runs (blue and yellow illumination) per session. |
| Behavioral performance measures | a/n |

## Acquisition

| | |
|---|---|
| Imaging type(s) | functional, anatomical |
| Field strength | 11.7T |
| Sequence & imaging parameters | a GE-EPI sequence with following parameters: 67 x 67 |

Sequence & imaging parameters: matrix, field-of-view 13.5 x 13.5 mm^2, TR/TE 1000/10.7 (ms), flip angle: 50◦, bandwidth: 333k(Hz), 31 coronal slices and slice thickness: 300 µm, and 690 repetitions

Area of acquisition: whole-brain excluding the olfactory bulb and cerebellum

Diffusion MRI: ☐ Used ☒ Not used

## Preprocessing

Preprocessing software: SPM12

Normalization: Linear and non-linear transformations were estimated between the anatomical images and the reference template using SPM12.

Normalization template: Templates for In vivo Mouse Brain, https://www.nitrc.org/projects/tpm_mouse

Noise and artifact removal: 6 movement artifacts are removed by multiple regressors

Volume censoring: n/a

## Statistical modeling & inference

Model type and settings: First level analysis carried out using general linear model (Mass univariate with random effects analysis)
Second level analysis carried out using permutation test (Mass univariate with random effects analysis)

Effect(s) tested: First level: Photostimulation blocks vs baseline
Second level: Wild-type mice vs. transgenic mice, Anesthetized vs. Awake

Specify type of analysis: ☐ Whole brain ☐ ROI-based ☒ Both

Anatomical location(s): Anatomical atlas derived from Allen Institute for Brain Science atlas.

Statistic type for inference (See Eklund et al. 2016): voxel-wise

Correction: permutation test with family-wise error cluster correction after probabilistic threshold-free cluster enhancement (TFCE), https://spisakt.github.io/pTFCE/

## Models & analysis

| n/a | Involved in the study |
| --- | --- |
| ☒ | ☐ Functional and/or effective connectivity |
| ☒ | ☐ Graph analysis |
| ☒ | ☐ Multivariate modeling or predictive analysis |
